# Selective and mild oxidation of α-hydroxyl groups in lignin by 2-iodoxybenzoic acid

**DOI:** 10.3389/fbioe.2025.1654739

**Published:** 2025-09-09

**Authors:** Fei Jiang, Zhiming Dong, Xiaoli Lou, Zhuonan Zhang, Li Shuai, Chengke Zhao

**Affiliations:** College of Materials Engineering, Fujian Agriculture and Forestry University, Fuzhou, China

**Keywords:** biomass, lignin, selective oxidation, structural modification, lignin condensation, lignin depolymerization

## Abstract

Secondary benzyl alcohols (α-OH) in lignin β-O-4 linkages are reactive sites that can trigger undesirable condensation reactions during lignin valorization processes. The selective oxidation of α-OH groups can effectively inhibit lignin condensation reactions, thereby promoting lignin depolymerization. Meanwhile, this approach increases the functionalities, which is beneficial for the design of lignin-derived functional materials. In this study, we report a chemoselective oxidation method using 2-iodoxybenzoic acid (IBX) to selectively convert α-OH groups into ketones. The reaction proceeds under ambient conditions with green DMSO as the sole solvent. Oxidation of β–O–4 model compound (veratrylglycerol-β-guaiacyl ether, VG) revealed exceptional selectivity of this oxidation process, achieving 92% substrate conversion with 90% yield of the corresponding α-ketone product after 48 h. When applied to cellulolytic enzyme lignin, this protocol significantly increased the α-ketone content from 1.2% to 20.5% (relative to total interunit linkages), as identified and quantified by 2D HSQC NMR. The straightforward α-OH oxidation protocol demonstrates significant potential for precise lignin structure modification and rational design of lignin-derived functional materials in sustainable polymer systems.

## 1 Introduction

Lignocellulosic biomass, composed primarily of three polyol biopolymers—cellulose (40%–60%), hemicellulose (15%–35%), and lignin (20%–30%) (Deng et al., 2023; Xie et al., 2024), has garnered extensive attention as a renewable resource for producing sustainable chemicals and functional materials (Yamada et al., 2013; Zhu et al., 2023). Selective oxidation of lignocellulosic biopolymers to introduce highly reactive functional groups, such as ketone, into their polymer structures facilitates their downstream processing, selective modification, and grafting with other polymers (Yu et al., 2019; Yuan et al., 2022).

Lignin is a complex aromatic polymer composed of phenylpropyl structural units (Dessbesell et al., 2020; Huang et al., 2020; Li et al., 2020; Sudarsanam et al., 2020; Zhang and Wang, 2020). Among its inter-unit linkages, the β-aryl ether (β–O–4) bond is the most predominant, accounting for over 50%–80% of the total chemical connections (Rahimi et al., 2013; Sun et al., 2018; Panovic et al., 2019; Sternberg et al., 2021). The β-aryl ether structure features a primary alcohol at C_γ_ position (γ-OH) and a secondary benzyl alcohol at C_α_ position (α-OH). Selective oxidation of α-OH to carbonyl group in β–O–4 unit can effectively inhibit undesirable condensation reactions (Rahimi et al., 2013; Lan et al., 2018; Lan et al., 2019) and promote the depolymerization of lignin by weakening the ether bond. Besides, the oxidation of the α-OH in β-aryl ether generates carbonyl group, thereby enhancing the functionality of lignin polymer and providing a specific reaction site for chemical modification, such as nucleophilic addition with various reagents (e.g., amines or alcohols). Existing oxidation systems, including the processes by using 2,3-dichloro-5,6-dicyano-1,4-benzoquinone (DDQ) (Lancefield et al., 2015), TEMPO (Rahimi et al., 2013), and N-hydroxyphthalimide (NHPI) (Zhang et al., 2017) demonstrated effectiveness in the selective oxidation of α-OH in β–O–4 units. However, these reaction systems are often complex, typically requiring the use of co-catalysts or elevated reaction conditions (Vangeel et al., 2018).

2-lodoxybenzoic acid (IBX) is a representative high-valence organic iodine reagent widely utilized for selective oxidation of reactive alcohols. The oxidation reaction is typically conducted in polar solvents such as dimethyl sulfoxide (DMSO) to stabilize the intermediates. Additionally, owing to its single-electron transfer mechanism, IBX-mediated oxidation generates fewer by-products and is easily operated (Maity et al., 2018). These versatile characteristics prompted us to explore the potential of IBX for selective oxidation of polyol-containing lignin biopolymers.

In this work, IBX was employed as an oxidizing agent for the oxidation of β–O–4 model compound and lignin in DMSO under ambient conditions. The results demonstrated that IBX selectively oxidized the α-OH groups to α-ketone groups in β–O–4 units while maintained the structural integrity of γ–OH groups. This study presents a mild and facile method for the selective oxidation of lignin polymers, offering potential for precise lignin structure modification and rational design of lignin-derived functional materials.

## 2 Materials and methods

### 2.1 Chemicals and materials

2-lodoxybenzoic acid (97%), guaiacylglycerol-β-guaiacyl ether (≥97%), pyridine (≥99.5%), N,O-bis(trimethylsilyl)trifluoroacetamide (BSTFA, 99%), acetic anhydride (≥99%), dimethylsulfoxide-d6 were purchased from Shanghai Macklin Biochemical Technology Co., Ltd. Dichloromethane, sodium hydroxide (97%), veratrylglycerol-β-guaiacyl ether (VG, 95%), ethylacetate (99.5%), sodium acetate trihydrate (99%), dimethyl sulfoxide, *n*-phenylpentane (internal standard, 99%) were purchased from Shanghai Aladdin Biochemical Technology Co., Ltd. Sodium chloride (99.5%), was purchased from Beijing Inno Chem Science & Technology Co., Ltd. Viscozyme were purchased from Novozymes. Acetic acid, 1,4-dioxane, tetrahydrofuran (THF), and ethanol were all analytical reagent grade. The water used in all experiments was ultrapure (18.25 MΩ). All chemicals and reagents were used without further purification.

### 2.2 Oxidation of β-O-4 model compound

VG or GG (5 mg) and IBX (10 mg, 2.2–2.4 equivalent) were dissolved in 1 mL of DMSO. After reaction for a period of time, 1 mL of internal standard (3 mg/mL n-pentylbenzene) was added into the solution. The mixture was then transferred into a separating funnel charged with 10 mL of saturated NaCl solution. After acidifying with diluted hydrochloric acid (pH < 3), the mixture was extracted with ethyl acetate (3 × 5 mL). The combined organic phase was dried over anhydrous sodium sulphate, and concentrated under reduced pressure. The obtained crude product was dissolved in 5 mL of dichloromethane for GC-FID and GC-MS analysis. 10 μL of the crude product solution was placed into the sample bottle charged with 80 µL of BSTFA and 100 µL of pyridine. The mixture was then heated in an oven at 50 °C for 1 h. The sample is tested by a GC-MS and a GC-FID for qualitative and quantitative analysis, respectively. The product yield was calculated using the following Equation 1 (Yang et al., 2023):

$$Y_{product}=\frac{n_{phenylpentane}\times S_{product}\times C_{phenylpentane}}{n_{VG}\times S_{phenylpentane}\times C_{product}}\times 100\%$$ (1)

Y_product_—the yield of the product (%);
n_phenylpentane_ (mmol)—the molar amount of the internal standard (phenylpentane);
S_product_—the peak area of product in the GC–FID chromatogram;
C_phenylpentane_—the effective carbon number of phenylpentane;
n_VG_ (mmol)—the molar amount of the internal standard (VG);
S_phenylpentane_—the peak area of phenylpentane in the GC–FID chromatogram;
C_product_—the effective carbon number of product.

After oxidation, the α-oxidized β-O-4 model compound (α-OVG) was separated using a preparative liquid chromatograph (Sepabean SP-2, Santai Technologies, Changzhou, China) equipped with a UV detector and a silica gel chromatographic column. An eluent consisting of ethyl acetate (EtOAc)/petroleum ether (1:3, v/v) was used for separation. Eventually, the crude α-OVG product was obtained with an isolated yield of 85%. The structure of the collected fraction was first confirmed by GC-MS and further characterized via NMR spectroscopy.

### 2.3 Cellulolytic enzyme lignin (CEL) isolation

Eucalyptus wood powder less than 40 mesh was milled by a Planetary Ball Mill for 48 h at 400 rpm. The ball milled wood powder (10 g) was then hydrolyzed in 400 mL of sodium acetate buffer (pH = 5.5) containing 300 mg of cellulosic enzyme (Celluelast 1.5L) and 400 mg of plant fiber complex enzyme (Viscozyme L) at 50 °C for 48 h (two times). After enzymatic hydrolysis, the suspension was centrifuged and washed with deionized water (2 × 60 mL). The solid residual obtained after centrifugation was extracted with 200 mL of dioxane/water (96:4, v/v) for 12 h. After centrifugation, the solution was concentrated at 45 °C under reduced pressure. The concentrated solution (∼3 mL) was droped into 50 mL of acidified deionized water (pH = 2), and the deposited lignin was obtained after centrifugation followed by vacuum drying.

### 2.4 Oxidation of lignin

100 mg of eucalyptus CEL and 100 mg of IBX were dissolved in 2 mL of DMSO and reacted at room temperature for 12–48 h. After reaction, the solution was added to 100 mL of DCM for deposition of the lignin. The mixture was then filtered and the solid was washed with DCM (3 × 10 mL). The oxidized lignin was finally obtained after drying in a vacuum oven.

### 2.5 Gel permeation chromatography (GPC) analysis

To a reaction vial, 5 mg of lignin sample was mixed with 0.5 mL of acetic anhydride and 0.5 mL of pyridine and reacted at room temperature for 24 h. The reaction mixture was then concentrated under reduced pressure at 45 °C, with anhydrous ethanol continuously added to remove residual reagents. The acetylated lignin was dissolved in 2 mL of tetrahydrofuran (THF) to achieve a concentration of 2.5 mg/mL, filtered through a 0.45 μm PTFE membrane, and subjected to GPC analysis by using an Agilent 1,260 high-performance liquid chromatography (HPLC) connecting with a refractive index detector. Elution was carried out at a column temperature of 35 °C using THF as the mobile phase at a flow rate of 1.0 mL/min. Molecular weight parameters were determined relative to polystyrene standards.

### 2.6 2D HSQC NMR characterization

The 2D HSQC NMR spectra of cellulolytic enzyme lignin (CEL) and oxidized CEL (OCEL) were analyzed by 600 MHz NMR spectrometer (AVANCE NEO 600 Bruker). CEL or OCEL (∼80 mg) was dissolved in 0.5 mL of DMSO-d_6_ in an NMR tube. The 2D HSQC NMR spectra were acquired from 10 to 0 ppm in F_2_ (^1^H) with 256 data points and a 1 s recycle delay, and 160–0 ppm in F_1_ (^13^C) with 256 increments. Total scan number of 32 was employed.

## 3 Results and discussion

### 3.1 Oxidation of β-O-4 model compounds by IBX

**FIGURE 1:**
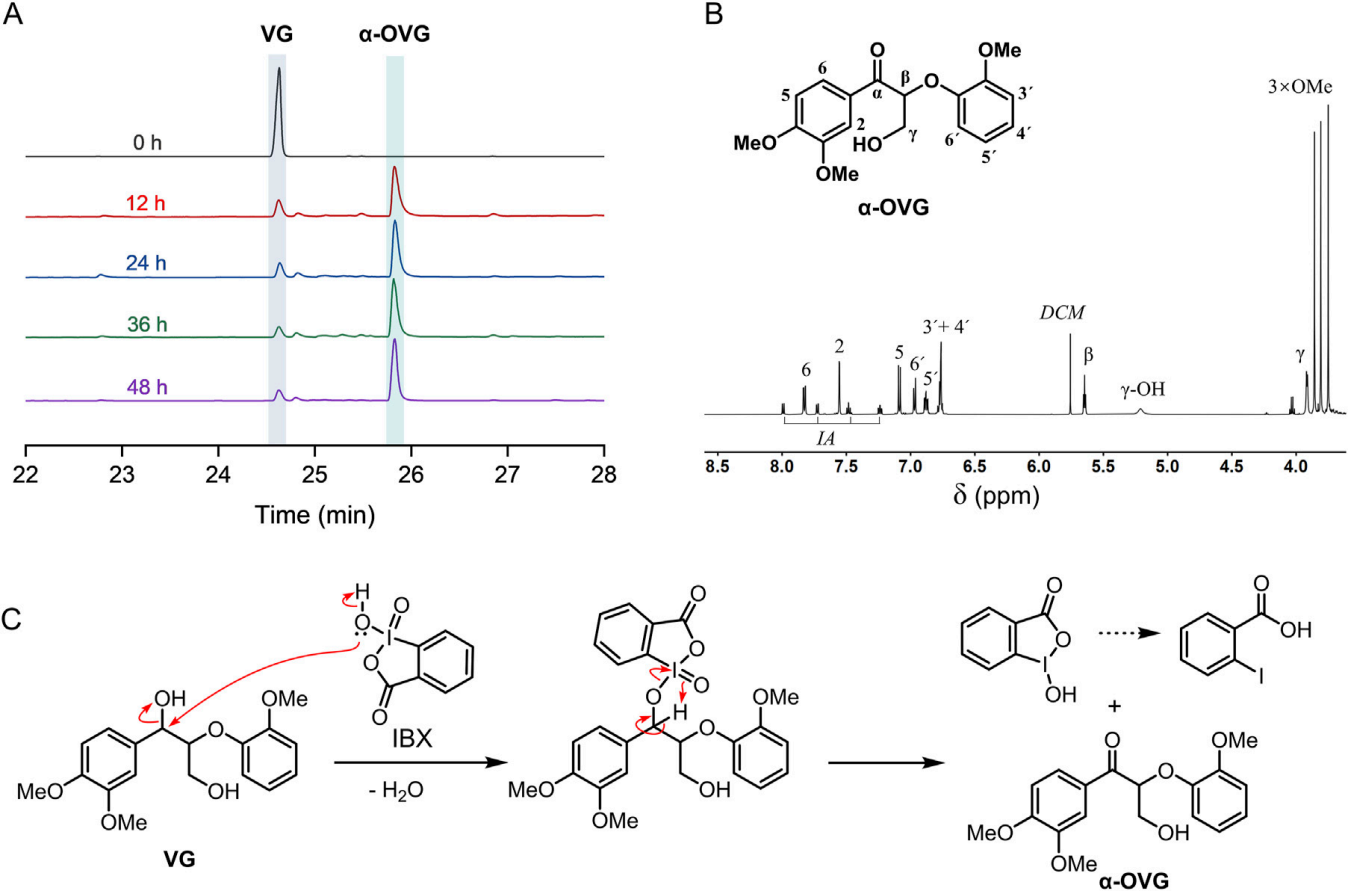
Oxidation of β–O–4 model compound (VG) by IBX. **(A)** GC chromatograms of the substrate (VG) and product (α-OVG); **(B)** ^1^H NMR of α-OVG. A traces of 2-Iodobenzoic acid (IA) is contained in the crude α-OVG product. **(C)** Proposed mechanism for oxidation of VG by IBX.

First, we evaluated the efficacy of IBX in oxidizing a β–O–4 model compound. The β–O–4 model compound, veratrylglycerol-β-guaiacyl ether (VG), was oxidized by IBX in DMSO solvent under ambient conditions. Post-oxidation, the resultant products were analyzed by GC-MS. The structure of main product was authenticated by mass spectrometry and ^1^H NMR after separation. In GC chromatograms, a typical product was identified at the retention time of 25.8 min (Figure 1A). Analyzing by mass spectrometry and ^1^H NMR (Figure 1B) confirmed its identity as the α-oxidized derivative of VG (α-OVG). γ-oxidized VG product (γ-OVG) was not identified from GC-MS analysis. This result demonstrates a high selectivity of IBX for oxidizing the second benzylic alcohol in the VG compound. The proposed mechanism for the oxidation of VG by IBX is depicted in Figure 1C. It is well established that the secondary alcohol at the benzylic carbon exhibits higher reactivity than the hydroxyl group at the γ-carbon, which accounts for the chemoselective oxidation occurring at the α-OH position.

To investigate the oxidation process of VG by IBX, 5 mg of substrate and 10 mg of IBX (∼2.3 equiv.) were mixed for reaction (Table 1, entry 1–4). As the reaction time was extended from 12 to 48 h, the conversion of VG increased from 81.6% to 92%, and the yield of α-OVG rose from 76.2% to 90.5%. However, when the amount of IBX was reduced to 5 mg (∼1.1 equiv.), both the VG conversion and the α-OVG yield remained low, even after a prolonged reaction time of 48 h (entry 5 in Table 1). These results suggest that the addition of approximately two equivalents of IBX is optimal for the efficient oxidation of VG. Furthermore, phenolic β–O–4 model compound (guaiacylglycerol-β-guaiacyl ether; GG) was employed as a substrate for oxidation. As shown in Table 2, only 3.3% yield of α-OGG was obtained. The major product observed was QGG. This result is attributed to the presence of a phenolic hydroxyl group and an ortho-methoxy group in GG. IBX preferentially oxidized the phenolic hydroxyl group and mediated the oxidative demethylation of the ortho-methoxy methyl group (-CH_3_). These results suggest that this oxidation process primarily targets the α-OH group within non-phenolic β-O-4 units.

**TABLE 1:** Oxidation of nonphenolic β–O–4 model compound (VG) by IBX.

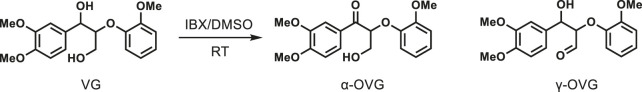						
Entry	VG (mg)	IBX (mg)	Time (h)	Conversion (%)	α-OVG (%)	γ-OVG (%)
1	5	10	12	81.6	76.2	ND
2	5	10	24	84.6	79.8	ND
3	5	10	36	90.8	88.2	ND
4	5	10	48	92.0	90.5	ND
5	5	5	48	48.6	44.5	ND

**TABLE 2:** Oxidation of phenolic β–O–4 model compound (GG) by IBX.

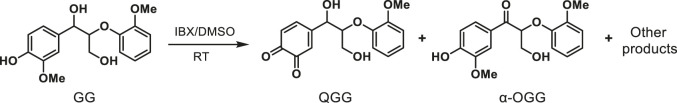					
GG (mg)	IBX (mg)	Time (h)	Conversion (%)	QGG (%)	OGG (%)
5	10	48	89.7	56.2	3.3

### 3.2 Oxidation of lignin polymer

Based on the successful oxidation of α-OH in β–O–4 model compound by IBX, we further investigate the effectivity of IBX for oxidation of lignin polymer. Eucalyptus cellulolytic enzyme lignin (CEL) was isolated and subjected to IBX oxidation with an equal mass (lignin/IBX: 1:1, w/w). The CEL and oxidized CEL (OCEL) were characterized by 2D HSQC NMR and GPC to elucidate the change in chemical structure and molecular weight.

**FIGURE 2:**
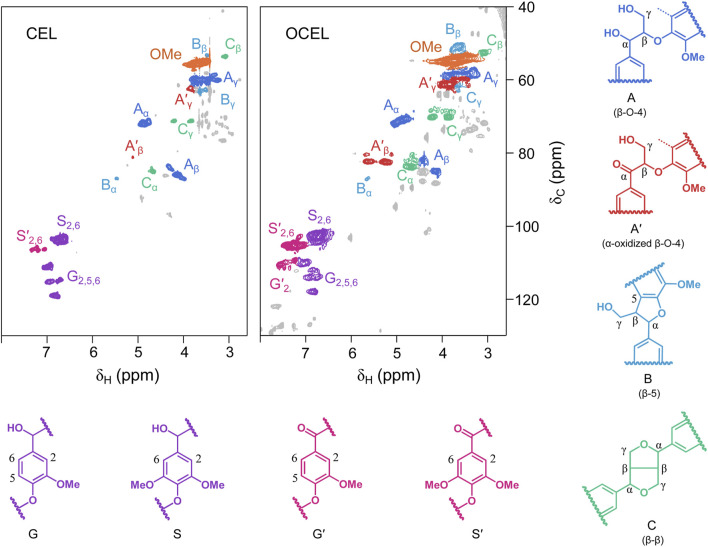
2D HSQC NMR spectra of eucalyptus cellulolytic enzyme lignin (CEL) and the CEL after IBX oxidation (OCEL).

**FIGURE 3:**
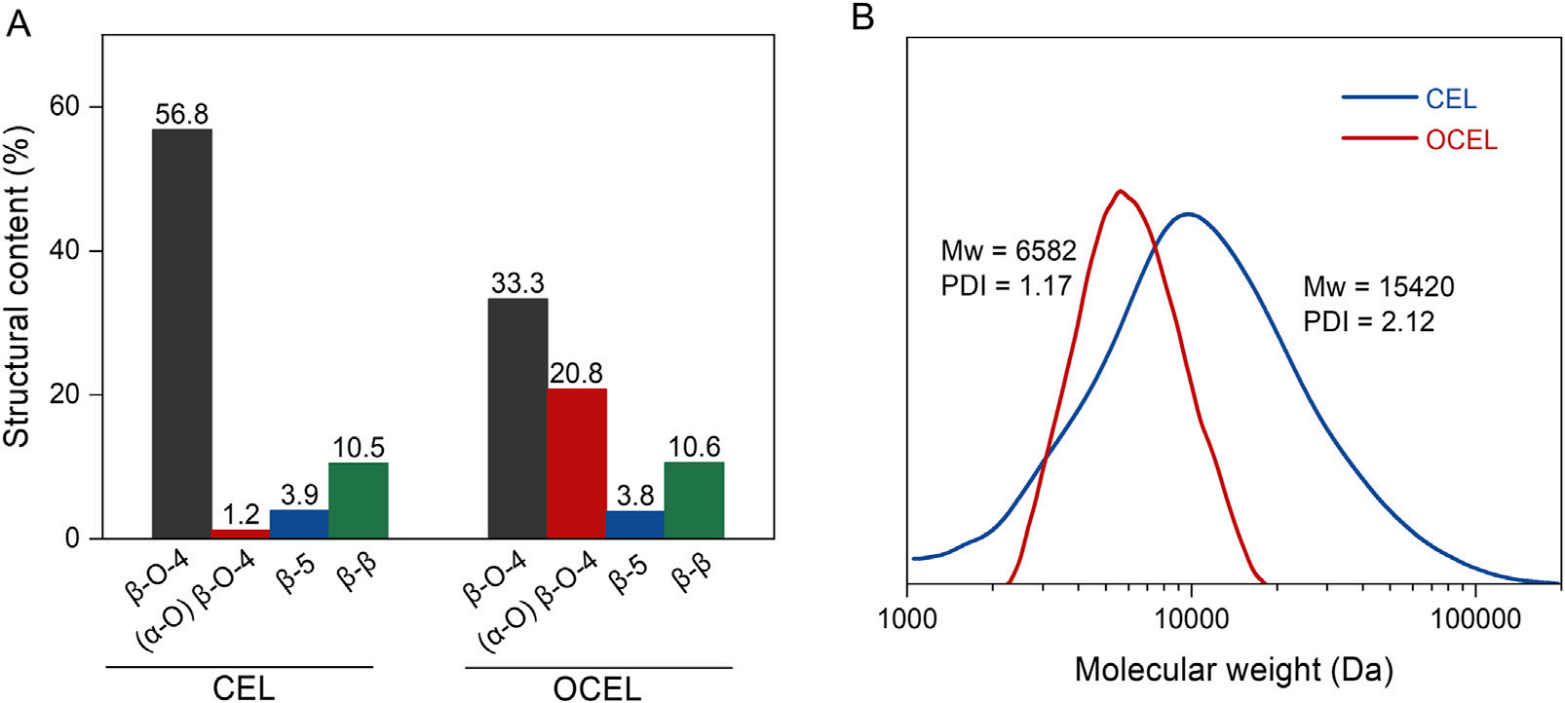
**(A)** The content of lignin structures in CEL and OCEL; **(B)** Molecular weight distribution of CEL and OCEL.

As shown in 2D HSQC NMR spectra (Figure 2), CEL consists of high content of β–O–4 linkage (56.8%), lower amounts of β–5 (3.9%) and β–β (10.5%) linkages, and trace of α-oxidized β–O–4 linkage (1.2%). After IBX oxidation, the content of β–5 and β–β remained nearly unchanged. However, the content of the native β–O–4 linkage significantly decreased. In the side chain region of the OCEL spectrum, a substantial number of α-oxidized β–O–4 structures (A′) were identified, with their C_β_–H_β_ and C_γ_–H_γ_ crosslinked signals appearing at 81/5.3–5.7 ppm and 62/3.7 ppm, respectively. Additionally, α-ketoyl aromatic ring structures (G′ and S′) were detected in the conjugated aromatic ring region of the OCEL spectrum. Quantitative analysis via 2D HSQC NMR revealed that the content of α-oxidized β–O–4 structures in CEL increased markedly to 20.8% after IBX oxidation (Figure 3A), with an oxidation yield of 34.5%. These results suggests that IBX can selectively oxidize the native β–O–4 units in lignin to form α-ketoyl β–O–4 structures. The lower oxidation efficiency in lignin compared to that in VG compound can be attributed to the presence of other linkages and free phenolic hydroxyl groups in lignin polymer, which can consume a portion of IBX.

The molecular weight distributions of CEL before and after oxidation were analyzed using gel permeation chromatography (GPC) to investigate the potential degradation of lignin during the oxidation process. As shown in Figure 3B, the oxidized product (OCEL), exhibited a lower molecular weight (weight-average molecular weight of 6,582 Da), compared to the unoxidized CEL (weight-average molecular weight of 15,420 Da). This reduction in molecular weight indicates that the IBX-mediated oxidation induces the degradation of lignin polymer. This degradation can be attributed to the formation of α-ketone groups during oxidation, which destabilize the β-aryl ether linkages in the lignin structure (Rahimi et al., 2013; Ren et al., 2017; Ji et al., 2023; Liu and Dyson, 2023), thereby rendering the polymer more susceptible to cleavage under the IBX oxidation conditions.

Overall, our findings demonstrate that IBX serves as a versatile oxidizing agent, capable of effectively oxidizing secondary benzyl alcohols to ketone groups in the β–O–4 units of lignin. Although the relatively high cost of IBX and stoichiometric oxidation of this method may limit its large-scale application, this method offers a mild and straightforward approach for the modification or functionalization of lignin materials, particularly in scenarios where precise chemical transformations are required.

## 4 Conclusion

In summary, we have successfully developed a mild and facile IBX-based oxidation method capable of selectively oxidizing of secondary benzyl alcohols in the β–O–4 units of lignin polymers without the use of toxic metallic catalysts. The method provides a mild and efficient strategy for the modification and functionalization of lignin materials. Its operational simplicity and selectivity make it a promising tool for advanced biomass valorization and functional material design.

## Data Availability

The original contributions presented in the study are included in the article/supplementary material, further inquiries can be directed to the corresponding authors.
